# A novel metadata management model to capture consent for record linkage in longitudinal research studies

**DOI:** 10.1080/17538157.2017.1364251

**Published:** 2017-11-06

**Authors:** Christiana McMahon, Spiros Denaxas

**Affiliations:** a University College London, Institute of Health Informatics, London, United Kingdom; b Farr Institute of Health Informatics Research, London, United Kingdom

**Keywords:** electronic health records, data linkage, consent, metadata, epidemiology, biomedical informatics

## Abstract

*Background:* Informed consent is an important feature of longitudinal research studies as it enables the linking of the baseline participant information with administrative data. The lack of standardized models to capture consent elements can lead to substantial challenges. A structured approach to capturing consent-related metadata can address these. *Objectives:* a) Explore the state-of-the-art for recording consent; b) Identify key elements of consent required for record linkage; and c) Create and evaluate a novel metadata management model to capture consent-related metadata. *Methods:* The main methodological components of our work were: a) a systematic literature review and qualitative analysis of consent forms; b) the development and evaluation of a novel metadata model. *Discussion:* We qualitatively analyzed 61 manuscripts and 30 consent forms. We extracted data elements related to obtaining consent for linkage. We created a novel metadata management model for consent and evaluated it by comparison with the existing standards and by iteratively applying it to case studies. *Conclusion:* The developed model can facilitate the standardized recording of consent for linkage in longitudinal research studies and enable the linkage of external participant data. Furthermore, it can provide a structured way of recording consent-related metadata and facilitate the harmonization and streamlining of processes.

## Introduction

Longitudinal health research studies provide a unique insight into the lives of people and are fundamental to investigating disease etiology and prognosis. Researchers are increasingly linking investigator-led studies, clinical cohorts, genomic datasets, and other administrative data sources together to produce enriched datasets that accurately reflect the longitudinal nature of diseases and capture the patient pathway through disease states.

**Table 1.** Thematic analysis of manuscripts.

Theme	Number of manuscripts
Analysis^21–32^	12
Comparison of models^33–47^	15
Consent aspects of secondary uses of data^48,49^	2
Development of a new model of consent/form^50–54^	5
Development of tools to assist consent process^55,56^	2
Discussion of a single model^57–60^	4
Establishing and/or improving participant understanding^4,61–68^	9
Other^69–80^	12

There are two types of record linkage: a) deterministic record linkage which involves integrating records using common unique identifiers (e.g. unique healthcare or social security identifiers) found in multiple disparate datasets and b) probabilistic record linkage where the probability that two disparate records relate to one individual is calculated. Informed consent is a key aspect of the health research process and to enabling record sharing and linkage. When obtaining consent, individuals can consent for themselves or on behalf of another; for example, a legal guardian/parent consenting on behalf of an infant. In such scenarios, the extent to which a legal guardian/parent consents on behalf of a child lessens as the child matures and their autonomy increases.^1^

When collecting research data as part of research studies, researchers handle consent in different ways as no common standard approach exists. When linking data for participants located across different data sources, these different consent models and approaches must be harmonized and aligned before data custodians and relevant governance bodies provide approval. By simplifying and standardizing the consent process, there is the potential for researchers to better understand the extent to which consent could have been given by participants and under which conditions.^3^ While limited guidance on the format and wording of consent is available, information quickly becomes out of date given the dynamic nature of the underlying ethical and legal frameworks and there is a lack of standardized methods to record the process and capture accurate data.^2,4,5^ These inconsistencies between consent models often cause substantial delays in the data linkage process due to delays in obtaining approval or may completely halt the process if no consensus is reached making the standardization of the process crucial.

The aim of our work was to create and evaluate a novel metadata management model to enable the capture of consent metadata associated with record linkage in longitudinal health studies in a systematic and standardized manner. Specifically, we sought to: a) systematically identify and review the current methodologies for recording consent; b) comprehensively review key consent elements of contemporary longitudinal health research studies; and c) design, implement, and evaluate a novel metadata model for capturing consent for record linkage.

Recording consent metadata in a structured and consistent manner can enable the development of tools and methods to facilitate the automated or semi-automated generation and sharing of consent metadata amongst stakeholders such as researchers, ethics committees, and data custodians. Consequently, this can pave the way toward streamlining the process across the scientific community and reducing the barriers which cause delays. The focus of our research has not been to determine the best practice from the viewpoint of ethical bodies or data custodians due to the dynamic nature and temporal changes in the underlying legal and ethical frameworks, but to provide a structured manner in which stakeholders can capture, document, and track the consent process. While the focus of this manuscript has been on longitudinal health research studies, many of the challenges associated with the consent process apply to clinical studies performed in healthcare settings and the proposed methodology can be adapted to apply in that context.

## Methods

### Literature review

We performed a cross-disciplinary literature review in January 2016 to determine the current state-of-the-art and identify literature describing the development of metadata management models to capture consent for record linkage. We used PubMed, Ovid, Scopus, The Cochrane Library, JSTOR, ACM Digital Library, Lecture Notes in Computer Science, Web of Science, Inspec, Google, Google Scholar, Intute, and forward citation tracking^6^ to the source literature. The search terms used were: ‘consent forms’, ‘longitudinal studies’, ‘record linkage’, ‘informed consent’, and ‘consent models’. To be included in the review, the results had to be available in English and either be openly accessible or accessible using institutional credentials. We synthesized the results by identifying the primary theme of the manuscript and categorized results accordingly.

### Metadata model implementation

In order to inform the design and development of our model, we comprehensively analyzed the questions and accompanying text of 30 consent forms from nine longitudinal research studies identified through a combination of desk research and engaging with stakeholders. Included studies are investigator-led, longitudinal consented research studies with a substantial record linkage component: ALSPAC^7^, Born in Bradford^8^, British Household Panel Survey^9^ (BHPS), Health Survey for England^10^, Life Study^11^, Millennium Cohort Study^12^ (MCS), Scottish Health Surveys^13^, UK Biobank^14^ and Understanding Society.^15^ We qualitatively analyzed the consent forms and identified relevant consent elements that directly informed the creation of the model’s metadata elements. The elements were subsequently grouped according to themes which were collated inductively and iteratively. By adopting an object oriented modeling approach we constructed the final metadata model.

### Metadata model evaluation

We evaluated the developed model by iteratively applying/adjusting it to consent forms from case studies (selected with the same criteria as before) in order to quantify its fit for purpose and critically appraise it. The studies used in the evaluation phase were: the English Longitudinal Study of Ageing (ELSA)^16^, the Canadian Longitudinal Study of Aging (CLSA)^17^ study, and Growing Up In Australia: The Longitudinal Study of Australian Children (LSAC).^18^

### Critical appraisal of DDI 3.2

We additionally mapped the identified metadata elements from our model to Data Documentation Initiative 3.2 (DDI 3.2) elements in order to critically appraise its fitness for recording consent elements in health research studies. The DDI is an XML-based metadata standard and was designed and developed primarily to describe social sciences research data. The standard is schema-based and currently there are two versions both incorporating Dublin Core elements: DDI-Codebook (DDI-2) and DDI-Lifecycle (DDI-3). DDI-3 encourages a more real-time approach to marking up metadata for long-term studies and provides mechanisms for metadata comparison. This was to determine the extent to which consent for record linkage in longitudinal studies may be recorded using the prevailing existing metadata standard. During our appraisal, we identified potential required extension which will be discussed.

## Results

### Literature review

**Figure 1.**
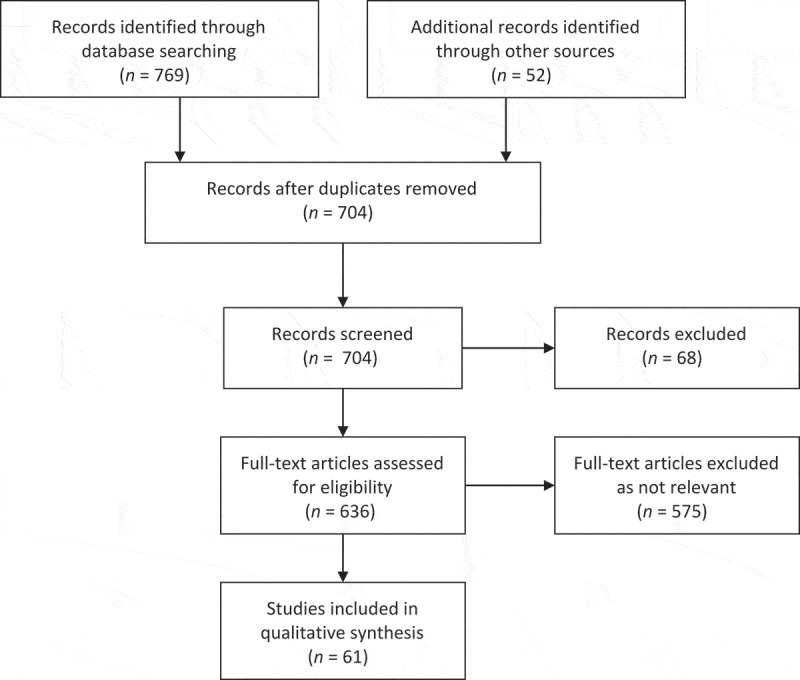
Literature review PRISMA flow diagram.

We identified and reviewed 61 manuscripts (Figure 1) and categorized them into themes inductively (Table 1) and iteratively: a) analysis – to either improve or establish knowledge; b) comparison of models – comparison of different types of consent; c) consent aspects of secondary uses of data – discussion of consent in the research context; d) development of tools to assist consent process – development of methods to assist with management; e) discussion of a single model – discussion of a single method of requesting consent; f) establishing and/or improving participant understanding – development and/or discussion of methods/tools to improve patients’ and participants’ understanding; and g) development of a new model/form and h) other – anything that does not fall under any other category.

### Metadata model implementation

Through qualitative analyses of consent forms and their associated patient information leaflets (where applicable) from the current longitudinal health research studies and stakeholder engagement, we identified and extracted the entities involved in the consent process. These formed the basic components of our metadata model for capturing consent in longitudinal studies: a) people, b) consent form, c) personal records, and d) informational material. We provide additional information on each component below.

#### People

We identified and extracted the common demographic details requested such as full name, dates and signatures of those consenting, statement of whether consent was on behalf of a child, confirmation of understanding statement and details of how to withdraw consent. The people most commonly involved in the consent process were identified as the person consenting (interviewee), and in certain situations, the person for whom consent is given if this is different from the interviewee. Others include the interviewer or staff member, as referred to in UK Biobank, teacher in the MCS, and General Practitioner (GP) in Life Study.

#### Consent form

We analyzed the composition and flow of information of the consent forms and identified a similar basic pattern used across all forms: demographic details, questions/statements of consent and then confirmation through the signing, dating, and printing of name on the form.

#### Personal records

Unsurprisingly, we identified that the most common type of record subjects were asked to consent for linkage was health and the least frequently used was for obtaining a participant’s criminal records. We also identified that in the consent forms for the Born in Bradford and Life Study, consent was requested to allow future contact with the participants. The longevity of such studies must be matched with consent models reflective of this and so being able to record this kind of information was critical to the development of our model.

#### Informational material

The final component focuses on the additional informational material such as leaflets which accompanied consent forms. Informational material is often used to describe the consent process, the flow of data and any regulatory or ethical considerations that are associated with participating with a study. In all studies, the material also contains information on how to withdraw consent from the study. Rarely, such as in the case of the ALSPAC study, the accompanying information describes how data linkage works and provides case studies of where record linkage has been used in the past and the benefits obtained from it.

### Metadata model evaluation

We evaluated the model by iteratively applying it to the three test cases randomly selected from the original pool of identified research studies (ELSA, CLSA, and LSAC) and documenting adjustments made to encapsulate all information from each study.

#### Person

We applied the model to the first test case, ELSA, and found that the model provided the metadata elements needed to record information about the interviewee and the interviewer. However, the model lacked elements to record when the consent form was completed; this indicated that the model needed revising to enable recording of this information. In terms of recording confirmation of understanding, we were able to use the ‘Confirmation of understanding’ attribute as part of the ‘Person’ element. This is a key aspect of our model as the need for a standardized approach to recording this kind of information is needed and this section of the model addresses this current unmet need. We then applied the model to the second test case, CLSA. We found that the metadata relating to the people involved could be recorded using the ‘Person’ element and the child elements, ‘Non-professional’ and ‘Professional’. In having two separate child elements, we were able to reduce the number of repeating attributes in the model through the use of inheritance whilst enabling ourselves to distinguish between the different types of people involved in the consenting process – ‘professional’ e.g. principal investigator and ‘non-professional’ e.g. interviewee. Following application of the model to the second test case, we decided to create a new element, ‘date of completion’, and that this should be moved, and joined to, the ‘consent form’ element. In doing so, we were able to group together all the elements relating to the consent form itself to help users better navigate through the model. The final test case used to evaluate the model was the LSAC study. Having iteratively applied the model to the previous test cases and made changes, we were able to record information such as ‘confirmation of understanding’ successfully.

#### Consent form

We firstly applied the model to ELSA to test how well our model could record information relating to the consent form’s composition. We found that use of elements such as ‘Academic institution’ enabled us to record detailed information, particularly since this element inherits the ‘Ethics approval reference’ and ‘Organization name’ attributes from the ‘Organization’ element. In using object-oriented modeling techniques, we were able to harness the advantages of a typically computer science technique for life sciences research. Following this initial test, we identified areas for improvement. For example, the ELSA consent form contains a set of instructions detailing what to do with the completed form – one copy is retained by the participant and the other is returned to the office. The model does not contain an element to record this information and so an additional element was needed. We altered our model accordingly, by adding ‘Instructions for next steps’, and applied the revised version to the second test case. The results of the second test, using the CLSA consent form, showed us that by adding the new element, we were then able to record more detailed information about this aspect of the consent process. The results of the second test also demonstrated that by harnessing the element such as ‘Questions’, as composed of ‘Logic’, ‘Responses’, ‘Purpose’, forming a part of the ‘Data collection’, we were able to record the questions and question logic of the consent form in detail. This is important as being able to identify and record the minutiae around this aspect of the consent process can potentially give stakeholders greater support in determining the scope of consent. The model was then reapplied to the final test case, LSAC. Here, we were able to test how well the model could, for example, record introductory information. Recording this information involved use of the ‘Aim’ and ‘Undertakings’ elements which are a part of the ‘General’ elements.

#### Personal records

Our model contains six different types of record: ‘Economic’, ‘Education’, ‘Legal’, ‘Family’, ‘Mobile phone usage’, and ‘Health’. We applied the model to the first test case, ELSA, and found we were able to record previous hospital visits and treatments through the ‘Health’ element and one of its child elements, ‘Past’. In having a child element ‘Past’, in addition to two others, ‘Present’ and ‘Future’ we were able distinguish between these different events. In terms of recording economic information for example, we used the ‘Economic’ elements with attributes: ‘Benefits claims’, ‘NI contributions’, and ‘Tax’. However, to enhance the model further, we decided to convert these attributes into separate elements which, when combined, create the ‘Economic’ element. We made this change to simplify the recording of this kind of information; in terms of cardinality, there would be no restrictions on the number of times elements can be used. We then applied the model to the second test case and were able to record health related information using the existing ‘Health’ element’. For the third test case, the LSAC study, we again used the ‘Health’ element but also used the ‘Persons’ element in addition to sufficiently record this information.

#### Information document

The results of the application to the ELSA study consent form demonstrated that to record different kinds of informational documents, the parent element, ‘Participant Information document’ and the child element ‘General’ needed to be combined to form a new element, ‘Informational document’ with ‘accessibility’, ‘audience’, and ‘type’ as attributes. This enabled the recording of, and differentiation between, the different types of informational document. However, we were not able to locate additional informational material online for ELSA; we also experienced this problem for the CLSA study. Therefore, we decided to proceed to the third test case, LSAC, to continue testing this aspect of the model. During the final test, we were able to use the newly-created ‘Informational document’ to specify the document type – in this case it was the corresponding information sheet. In making this change to the model, we reduced the total number of elements whilst increasing scope to record metadata relating to the different kinds of informational document. This is an advantage of using a formalized modeling technique as changes were made quickly without impacting the rest of the model. We then recorded the description of the study using the ‘Study’ element with attributes, ‘Aims’, ‘Contact details’, ‘Funding bodies’, ‘Objectives’, and ‘Reviewers’.

### Critical appraisal of DDI 3.2

We critically evaluated the DDI 3.2 metadata standard by assessing its feasibility to map and record each consent element from the basic components of our metadata model.

#### People

Half of the consent metadata elements were directly mapped to DDI elements. General metadata elements such as full name, location, nationality, contact details and date of consent can be mapped directly using the ‘FullName’, ‘LocationName’, ‘Country’, ‘TelephoneNumber’, ‘Email’, and ‘Date’ elements, respectively, allowing for the relatively accurate reflection of the people involved in the consenting processes. However, a fundamental aspect of consenting process associated with longitudinal studies is the possibility for the interviewee to consent on behalf of another person; for example, a mother consenting on behalf of her infant or a legal guardian consenting on behalf of somebody who is unable to provide consent on their own. We were unable to accurately capture and reflect this in DDI. Stakeholders could potentially use the ‘Note’ element along with another element to record this information. While this approach works in the short term, as the infant matures, and they become more able to assent/dissent, DDI lacks the necessary mechanisms to record this level of interaction in detail. Additional elements are needed in DDI to enable the recording of these critical pieces of metadata.

#### Consent form

We were able to map almost all of consent metadata elements to DDI elements. This process was fairly straightforward and we found that the majority of elements could be mapped directly. This is due to the fact that consent forms are a type of survey instrument and this is exactly what DDI is designed to capture. In some cases, such as responses, we identified two DDI elements which could be potentially used to record metadata – text can be recorded using ‘ResponseText’; or stakeholders could select a response from a predetermined, named list and use a combination of, ‘CodeList’, ‘CodeListName’, and ‘CodeListReference’ DDI elements. Nevertheless, DDI again lacks the elements needed to record the minutia. For example, we found that ‘Undertakings’ and ‘Confirmatory information’ could not be mapped directly to a DDI element. Again, while a potential work-around solution would be to use the ‘Note’ element to hold the necessary information and then attach this to another maintainable object, this is not a scalable approach and new DDI elements are required to accurately capture all the consent form elements required.

#### Personal records

All metadata elements related to personal records were mapped to DDI elements. For example, we were able to group together organizations and assign a group name. Here, the element ‘CodeListGroup’ can be used to specify the name of the group; while ‘CodeList’ will enable stakeholders to record the possible clinical terminologies used such as the International Classification of Diseases or SNOMED-CT. DDI 3.2 has built-in mechanisms to successfully record code lists and categories so the process of providing links between these elements was relatively straightforward. Other areas in which the DDI provided the necessary mechanisms to map directly included location – ‘LocationName’. However, the results of this analysis demonstrated that a direct link from elements associated with, ‘Treatments and management of conditions’ and ‘Tests and assessments’ to elements in DDI was not possible. Having access to this kind of metadata in a standardized and simplified format is key to supporting stakeholders in determining, use of health services (part of the current treatment and management of conditions) and rights to results (part of tests and assessments).

#### Informational material

We were able to map roughly half of consent metadata elements to DDI elements which can successfully record information about the study and study objectives using the ‘Citation’ element. Other elements such as funding bodies can also be mapped directly using the, ‘FundingInformation’ element. Another advantage of using DDI is that lifecycle events, such as a participant withdrawing their consent can be recorded, and mapped relatively easily. Here, a combination of ‘EventType’ and ‘LifecycleEvent’ would enable stakeholders to record this information in a clear and standardized manner. This is very important as the boundaries of consent have a direct impact on researchers wanting to use certain research data. DDI also enables stakeholders to create multiple lifecycle events. This can be used to the stakeholders’ advantage as they can record any additional relevant events in a systematic and robust manner. However, stakeholders are again restricted in recording details specific to consent for record linkage. For example, there are no elements in DDI which can be mapped directly enabling the recording of, use of biological samples and how these will be acquired. Being able to record, and have access to, this kind of metadata is important to informing potential secondary users of which biological samples could be used as part of their further analyses of the research data. Furthermore, having access to this kind of metadata can also help other stakeholders, such as potential participants understand what could be requested of them should they partake in the study.

## Discussion

During the initial literature review and following the thematic analysis of identified manuscripts, we were unable to identify any previous research focusing on the development of a metadata management model to record consent for record linkage in longitudinal health research studies. The search terms we used were specific to public health and epidemiological research and not readily used in other domains such as computer science. Therefore, possible differences in use of controlled vocabularies and terminologies may have negatively impacted our searching for literature. Additionally, as a result, our review did not include a body of methodological research on consent as it tends to focus on individual factors influencing the willingness of survey respondents such as, for example, the placement of wording on a consent form and was deemed to be outside the context of our study. Nevertheless, the literature review suggested that research into the development of consent models to record informed consent for record linkage in longitudinal studies is limited and merits further research.

The use of an object oriented approach to the design of our model, particularly *inheritance* and *aggregation* enabled us to reduce data repetition and reduce the overall model complexity and size. By using inheritance, multiple elements could share attributes. For example, in the consent form section using the third test case, in having a generalized parent element of ‘Organization’, attributes such as ‘Organization name’ are inherited by every child element helping to produce a simplified model capable of recording low level detail. The use of aggregation enabled us to specify the elements needed to compose metadata relating to the questions (using the ‘Questions’ element), or in this case, the three statements. This is an advantage of using object oriented modeling techniques to design and develop the model. As part of section D, an example is provided describing potential information that may be accessed. This may be captured using the ‘Research’ element as part of the ‘Informational document’ element.

Given the increasing costs of primary data collections associated with longitudinal health studies, a recent direction is to deploy a mixed-mode design i.e. involving a combination of offline paper-based and online form-based data collection instruments. In the UK for example, the National Child Development Study (NCDS) Age 55 Survey adopted a sequential mixed-mode design whereby study members were first invited to participate online, with non-respondents being followed up by telephone.^19^ The developed metadata model enables researchers and other stakeholders to record the manner in which consent is provided using the ‘Method of collection’ element (which is a part of the ‘Data collection’ element through aggregation). This provides users with the flexibility to distinguish between modes of consent (e.g. self-completion, interview-driven) given that methodological research in the area illustrates how self-completion has lower response rates than interviewer based models and can potentially introduce sample bias.^20^

A potential weakness of our approach and model lies in the fact that currently identifying metadata elements involves a qualitative analysis of the current consent forms and there is the potential for bias given the potential subjectivity of the process. Ideally, the entire process would be automated and identification of concepts would be through use of a predefined concept list, possibly structured using a pre-defined ontology, from which concepts may be selected and assigned. In having a fully automated process, this could potentially reduce human error and scope for bias. Furthermore, information documents could not be found online for ELSA and CLSA studies. This is a weakness in our approach to evaluating the model as this section of the model was not tested to the same extent as the other three sections. To enhance the model further, the ‘type of test’ and ‘storage of sample’ attributes of the ‘health’ element could be removed and placed in a new element entitled ‘biological samples’. Having a separate element for this information widens the scope for further extension and enables additional, element-specific attributes to be added such as name and site of labs. This could also potentially improve the extent to which dynamic consent may be captured as changes in the model would facilitate the recording of more low level detail e.g. the title of the particular test (such as the blood test) for which dynamic consent may be given – enabling consent to be given for the use of some tests and not others. Our model and approach focused on low-level metadata elements rather than wording or placement of elements on the consent form the focus of our research was not to determine what works best or how to maximize consent rates (as outlined above) but rather to provide an underlying structure and a mechanism to systematically capture and characterize that structure.

Finally, the results of the critical analysis indicated that whilst many DDI 3.2 elements may be harnessed to create standardized descriptions of consent for record linkage in longitudinal studies, the standard lacks the mechanisms needed to record low level metadata specific to epidemiological and public health research. It is in these areas in particular that the standard fails to provide the necessary mechanisms to record consent for record linkage using metadata elements effectively. For example, it was challenging to identify and select which elements could be used individually or together to record the interviewee’s thought process and reasoning when deciding the extent to which they would like to consent. It is having access to these details which could potentially better support researchers in undertaking analyses using record linkage. This is because these explanations could potentially provide the context with greater detail which researchers could potentially find useful. The advantage of DDI 3.2 sits very much in the opportunity for stakeholders to package together standardized instances of metadata in an interoperable format (XML) which can then be published as a ‘StudyUnit’. These instances may be entered into inter/national catalogues where they can be actively maintained. Subsequently, stakeholders such as potential secondary users, members of the public, and in addition to others, may view these metadata records to better inform themselves of the past and current longitudinal studies. Consequently, stakeholders can potentially have a greater understanding of what could be achieved if access to the data and/or biological samples was granted.

## Conclusion

Longitudinal health research studies are critical to investigating disease aetiology and prognosis and its impact across the life course. There is a distinct lack of standardized approaches to recording the metadata associated with consent to record linkage for such studies despite the fact that they are considered crucial and are widely used. In this study, we created and evaluated a novel metadata model to record consent for record linkage using metadata elements. The novel metadata management model can now assist researchers and other stakeholders in recording standardized descriptions of the consent process and facilitate the harmonization of the process across multiple research studies. Our long-term goal is to integrate the metadata model into a software tool which can assist stakeholders in recording consent for record linkage metadata using semi-automated processes and allow the sharing of computable consent-form definitions across the wider scientific community.

## Key messages

1. While obtaining informed consent from patients and participants is an important aspect of health research studies, as outlined by a systematic literature review, no common standardized approach for capturing and recording consent elements for health research currently exists.
2. A qualitative review of 30 consent forms from the existing longitudinal health research studies illustrated significant variation in terms of the type and manner of the information collected during the consent process.
3. The lack of systematic and standardized approaches to recording consent leads to significant delays in undertaking longitudinal health research studies, limits their secondary use by other researchers, and makes the linkage of multiple datasets challenging.
4. Standardized metadata models for capturing and recording consent across research studies can address some of these challenges and enable scientists to link disparate health datasets for research. Additionally, they can pave the way for streamlining the consent process and developing software applications to automate the process.
